# Selective G-Quadruplex DNA Recognition by a New Class of Designed Cyanines

**DOI:** 10.3390/molecules181113588

**Published:** 2013-11-04

**Authors:** Rupesh Nanjunda, Eric A. Owens, Leah Mickelson, Tyler L. Dost, Ekaterina M. Stroeva, Hang T. Huynh, Markus W. Germann, Maged M. Henary, W. David Wilson

**Affiliations:** 1 Department of Chemistry, Georgia State University, 50 Decatur St., Atlanta, GA 30303, USA; E-Mails: rnanjunda@gsu.edu (R.N.); eowens9@student.gsu.edu (E.A.O.); lmickelson1@student.gsu.edu (L.M.); tdost1@student.gsu.edu (T.L.D.); estroeva1@student.gsu.edu (E.M.S.); hhuynh8@student.gsu.edu (H.T.H.); 2 Center for Diagnostics and Therapeutics, Georgia State University, 100 Piedmont Ave SE, Atlanta, GA 30303, USA; 3 Department of Biology, Georgia State University, 495 Petit Science Center, 100 Piedmont Ave., Atlanta, GA 30303, USA

**Keywords:** G-quadruplex, cyanines, *c-myc*, telomere, end-stacking, halogenation, nuclear magnetic resonance, surface plasmon resonance, thermal melting

## Abstract

A variety of cyanines provide versatile and sensitive agents acting as DNA stains and sensors and have been structurally modified to bind in the DNA minor groove in a sequence dependent manner. Similarly, we are developing a new set of cyanines that have been designed to achieve highly selective binding to DNA G-quadruplexes with much weaker binding to DNA duplexes. A systematic set of structurally analogous trimethine cyanines has been synthesized and evaluated for quadruplex targeting. The results reveal that elevated quadruplex binding and specificity are highly sensitive to the polymethine chain length, heterocyclic structure and intrinsic charge of the compound. Biophysical experiments show that the compounds display significant selectivity for quadruplex binding with a higher preference for parallel stranded quadruplexes, such as cMYC. NMR studies revealed the primary binding through an end-stacking mode and SPR studies showed the strongest compounds have primary *K*_D_ values below 100 nM that are nearly 100-fold weaker for duplexes. The high selectivity of these newly designed trimethine cyanines for quadruplexes as well as their ability to discriminate between different quadruplexes are extremely promising features to develop them as novel probes for targeting quadruplexes *in vivo*.

## 1. Introduction

G-quadruplexes are higher order DNA structures formed by either intra- or intermolecular association of guanine-rich sequences into a stacked array of G-tetrads and stabilized by Hoogsteen hydrogen bonds as well as coordinated monovalent cations [1,2]. The formation and stabilization of these unique structural motifs are implicated in diverse biological functions such as gene regulation and chromosomal stability [3,4], therefore, these structures have garnered immense interest as a viable platform for the development of quadruplex-specific small-molecule drugs for therapeutic purposes [5,6]. Furthermore, with the recent exciting evidence showing the formation of G-quadruplexes during various stages of mammalian cell cycle *in vivo* as well as convincing evidence that small molecules can indeed induce and stabilize quadruplexes *in vivo*, these unique structures are undoubtedly novel and promising targets for the design and development of anticancer therapeutic agents using small molecules [7,8]. In fact, a wide range of quadruplex-specific small molecules have been reported so far with some compounds showing very promising biological activities [9,10,11].

**Figure 1:**
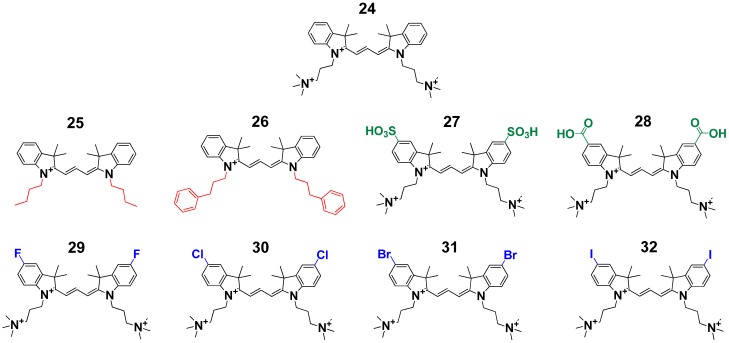
Trimethine cyanine analogs used in the current study. The modifications performed on the parent cyanine (**24**) are highlighted in different colors (compounds **25**–**32**). The detailed synthetic procedure is described in Section 4.1. All compounds have one positive charge on the cyanine system and most have charged alkyl amine substituents. The synthesis and characterization of the related pentamethine cyanine analogs (compounds **1**–**23**) are previously reported [18].

Cyanines are a class of small molecules that are being developed as potential G-quadruplex targeting agents in addition to their wide-range of applications as cancer imaging agents and nucleic acid stains [12,13]. The synthetic ability to structurally tailor cyanines as well as their many therapeutically favorable features, such as low toxicity, also renders them ideal candidates for their potential development as quadruplex targeting agents. In addition, the fluorescence properties of cyanines have been extensively developed in understanding the structural transitions associated with quadruplex folding/unfolding as well as in developing fluorescence agents for detecting different quadruplex conformers *in vitro* [14,15,16,17]. Development of quadruplex-specific fluorescence probes with reduced duplex affinity would significantly aid in targeting and visualization of quadruplex structures in an otherwise duplex-enriched genomic content. Previously we had reported a new class of indolenine scaffold based cyanine molecules with highly favorable quadruplex interactions [18]. The pentamethine linker between the two indolenine rings provided the needed flexibility for efficient stacking at the terminal tetrads of the quadruplex, whereas the dimethyl groups on the indolenine rings considerably reduced the intercalation or stacking of the cyanines in the duplex minor groove. The halogen substitutions at different positions on the aromatic core further increased their quadruplex affinity. The highly promising results of the pentamethine cyanines as quadruplex specific agents encouraged us to further explore the conformational space of cyanine molecules for increased quadruplex affinity and selectivity. In the current study, a series of cyanine molecules (Figure 1) with reduced methine linker length were synthesized to probe the effects on quadruplex binding while maintaining the dimethyl and halogen substitutions. Using a series of powerful biophysical techniques, we show that these newly designed trimethine cyanines exhibit more favorable quadruplex binding while maintaining a very low duplex affinity. Furthermore, the compounds exhibit higher selectivity for parallel-type quadruplex conformers. The results are highly promising and encouraging towards the development of cyanine-based small molecules as highly specific quadruplex targeting agents.

## 2. Results

### 2.1. UV-Thermal Melting Screening (Tm)

UV-Thermal melting is a rapid and robust screening technique to determine selectivity as well as to rank the ligand-induced relative stability of quadruplex-ligand complexes [19,20]. Preliminary *T*_m_ analysis of a synthetic set of symmetric trimethine cyanines containing different substituents on the indolenine rings (Figure 1) were conducted with a well-characterized telomeric quadruplex sequence (Tel22) and a control hairpin duplex sequence (AATT) that is quite favorable for detection of either minor groove or intercalative binding to duplexes. The increase in the thermal stability of the complexes upon ligand binding (∆*T*_m_) at different ligand-DNA ratios is listed in Table 1. A range of ligand-DNA mole ratios were employed to ensure saturation of binding sites on the quadruplex as well as duplex motifs. Drug-DNA ratios beyond 6:1 resulted in inconsistent melting curves in some cases due to possible aggregation. The trimethine-linked parent cyanine, compound **24**, exhibited a favorable increase in the thermal stability of the Tel22 sequence with increasing ratios. This increase in thermal stability of **24** with Tel22 is slightly improved compared to the *∆Tm* of a similar cyanine with a pentamethine linker reported previously (Table 1: compare ∆ *T*_m_s of **24** and **a**) [18]. Replacing the propyl-(trimethyl ammonium) groups on both indolenine rings of **24** with hydrophobic propyl-phenyl groups, **26**, slightly reduced the thermal stability of the Tel22 complex. A similar replacement with an n-butyl group, **25**, dramatically reduced the thermal stability of the complex compared to **24**. This suggests the importance of charge on the substituents to maintain favorable electrostatic interactions with the backbone of the anionic quadruplex motif. While retaining the propyl-(trimethyl ammonium) groups, various substitutions were made on the indolenine rings to probe their effect on quadruplex binding. Substitutions with either a sulfonato (–SO_3_H, **27**) or carboxylic acid (–COOH, **28**), which have negative charges under the experimental conditions, resulted in a minimal increase in the thermal stability of Tel22, probably due to unfavorable electrostatic interactions with the negatively charged backbone. In accordance with the design principles established in the previous studies to enhance quadruplex interactions by halogenation of cyanines, systematic halogen substitutions on the indolenine rings in the current trimethine series showed a highly favorable increase in the thermal stability of the telomeric quadruplex (Table 1, compounds **29**–**32**). Furthermore, the trimethine chloro (**30**) and bromo (**31**, Figure S1) analogs showed a more favorable increase in the thermal stability of Tel22 compared to the similar halogen-substituted pentamethine analogs (Table 1, compounds **b** and **c**). The trimethine-linker scaffold might be a better optimized system than the pentamethine-linker cyanines for more favorable interactions with a quadruplex unit. All the trimethine halogen derivatives, however, exhibited very similar increases in the thermal stability of the quadruplex motif suggesting a minimal effect of the size of the halogens.

**Table 1:** *T*_m_ changes of trimethine cyanine analogs with telomeric quadruplex (Tel22) and a control duplex (AATT).

Compound/Drug:DNA Ratio	Tel22				AATT duplex			
	1:1	2:1	4:1	6:1	1:1	2:1	4:1	6:1
**24**	2	4	9	14	0	1	1	2
**a**	-	2.1	4.6	8.7	0	0	0	0
**25**	1	1	1	0	ND			
**26**	1	2	4	11	0	2	2	2
**27**	2	1	1	1	ND			
**28**	1	1	3	7	1	1	2	3
**29**	3	8	12	16	0	1	1	2
**30**	3	7	15	19	0	0	1	1
**b**	-	3.1	8.9	13.2	0	0	0	0.5
**31**	1	5	14	24	0	0	0	0
**c**	-	2.5	15.6	-	0	0	0	1.1
**32**	3	7	17	21	1	1	3	3

The thermal melting values reported are an average of two independent trials and are reproducible within ±0.5 °C. Tm values of free Tel22 and AATT are 61 °C and 66 °C respectively. ND = Not Determined. a, b, c: ∆*T*_m_ values from previous studies for pentamethine analogs with the substitutions for **a**: X = H; **b**: X = Cl; **c**: X = Br and R = TMAB in all cases [18].

One of the important features observed with these cyanine molecules is their very low binding to duplex-DNA sequences (Table 1, ∆*T*_m_ values with a control sequence (AATT) that is quite favorable for minor groove or intercalation binding mode). The trimethine cyanines exhibited a very small-to-zero increase in the thermal stability with duplex-DNA even at relatively high ligand-DNA ratios. Representative UV-thermal melting curves with the Tel22 quadruplex and the control duplex are illustrated in Figure S1 for compound **31**, as comparative examples of what is observed with all compounds. The dimethyl substituents on the terminal indolenine rings, as predicted, provide the necessary steric hindrance and reduce any possible interactions with duplex-DNA. In line with the previous studies on pentamethine cyanines, these newly designed trimethine cyanines show an important step in further expanding the cyanine scaffolds as a novel quadruplex-targeting class of small molecules with reduced duplex-DNA selectivity.

### 2.2. Biosensor-Surface Plasmon Resonance Studies (SPR)

**Figure 2:**
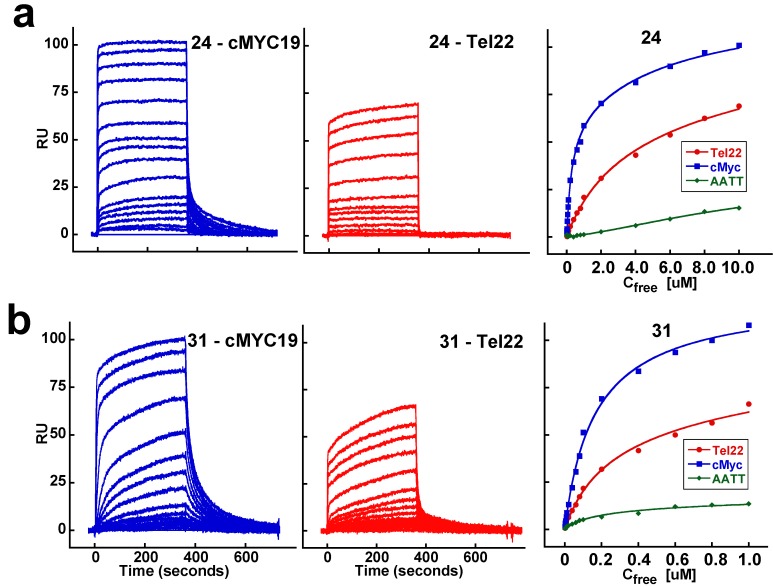
SPR sensorgrams and the steady-state binding plots for the parent cyanine (**24**, panel **a**) and the brominated analog (**31**, panel **b**) with Tel22 and cMYC19 quadruplexes and a control duplex (AATT). The injected concentration range for **24** is 10 nM–10 µM and for **31** is 10 nM–1 µM. The binding plots were obtained by fitting the steady-state response values (RU) as a function of free ligand concentration (C_free_) and fit to a two-site binding model. The estimated equilibrium binding affinities (*K*_1_ and *K*_2_) are reported in Table 2.

Biosensor-SPR is an effective technique to determine the equilibrium binding affinity and kinetics, as well as the stoichiometry and cooperativity of biomolecular interactions in real-time [21,22]. To determine the relative binding strength of the trimethine cyanine analogs, SPR experiments were performed with biotinylated quadruplex and duplex sequences. In addition to the Tel22 quadruplex, a well-characterized oncogenic promoter quadruplex sequence from *c-myc*, cMYC19, was also employed to determine if the compounds can exhibit any selectivity for different quadruplex architectures. Figure 2 shows representative SPR sensorgrams and the corresponding steady-state affinity plots for the parent cyanine **24** and the brominated analog **31** with both quadruplex-forming sequences. The sensorgrams show very fast association and dissociation of the compounds and were characteristic of all the analogs, except for compound **25** which did not exhibit any binding with either quadruplex sequences. The equilibrium binding affinities for all the analogs with both quadruplexes and a control duplex (AATT) were determined using a two-site binding model and are listed in Table 2. All the compounds exhibit reasonable binding to both quadruplex sequences with a primary strong binding (*K*_1_) followed by a weaker binding affinity (*K*_2_), ca. 10X lower. The halogenated analogs **29**–**3** showed impressive 5–15 times stronger binding affinities with the telomeric quadruplex compared to the parent compound (**24**, *K*_1_ = 3.9 × 10^5^ M^−1^) in agreement with the Tm studies. With the parallel cMYC19 quadruplex, compound **24** showed a more favorable 10 times increase in binding affinity compared to the telomeric quadruplex, however, the brominated analogs exhibited only a 2‒5 fold increase in the binding affinity. The anionic substitution analogs **27** and **28** showed a 2-fold increase in the binding affinity with Tel22 compared to the parent cyanine **24**, a trend that was not observed in Tm studies. In general, all high affinity ligands showed a higher preference for the parallel *c-myc* quadruplex over the hybrid telomeric quadruplex. The binding affinities for the duplex sequence were either too low (*K*_A_ < 10^5^ M^−1^) or could not be determined due to very low steady-state response values for the same concentration range (Table 2, AATT; Figure 2, green data points). SPR results, thus, conclusively show the selectivity of the trimethine analogs for quadruplex structures with different folding topologies as well as the selectivity over duplex sequences and in agreement with thermal melting studies. The two-site fitting model predicted two predominantly bound ligands per quadruplex unit probably stacked at the terminal tetrads (discussed below in NMR section), a common mode and stoichiometry of binding exhibited by a majority of quadruplex targeting ligands [9,23,24,25,26,27,28].

**Table 2:** Equilibrium binding constants of the trimethine cyanine analogs with Tel22 and cMYC19 quadruplex sequences obtained from SPR studies.

Compound	Tel22 (*K*_1_; *K*_2_) M^−1^	cMYC19 (*K*_1_; *K*_2_) M^−1^	AATT (*K*_1_) M^−1^
**24**	3.9 × 10^5^; 3.9 × 10^4^	3.8 × 10^6^; 1.4 × 10^5^	<10^5^
**25**	No Binding	No Binding	No Binding
**26**	1.3 × 10^5^; 1.7 × 10^4^	1.2 × 10^6^; 6.6 × 10^5^	<10^5^
**27**	8.2 × 10^5^; 5.8 × 10^4^	5.7 × 10^5^; 4.5 × 10^4^	<10^5^
**28**	8.3 × 10^5^; 6.1 × 10^4^	5.0 × 10^6^; 5.6 × 10^5^	<10^5^
**29**	5.9 × 10^6^; 3.9 × 10^5^	6.3 × 10^6^; 4.3 × 10^5^	<10^5^
**30**	2.7 × 10^6^; 2.9 × 10^5^	8.4 × 10^6^; 3.3 × 10^5^	<10^5^
**31**	5.5 × 10^6^; 4.5 × 10^5^	1.2 × 10^7^; 2.7 × 10^6^	<10^5^
**32**	2.1 × 10^6^; 2.2 × 10^5^	7.8 × 10^6^; 2.5 × 10^6^	<10^5^

The steady-state response obtained as a function of free ligand concentration was fitted to a two-site binding model to obtain *K*_1_ and *K*_2_ values. The binding constant values are reproducible with ±10%. No Binding: No sensorgrams were observed in the concentration range that was tested. In all cases, no significant duplex binding was observed suggesting a high selectivity of the ligands for quadruplexes.

### 2.3. Circular Dichroism Studies (CD)

**Figure 3:**
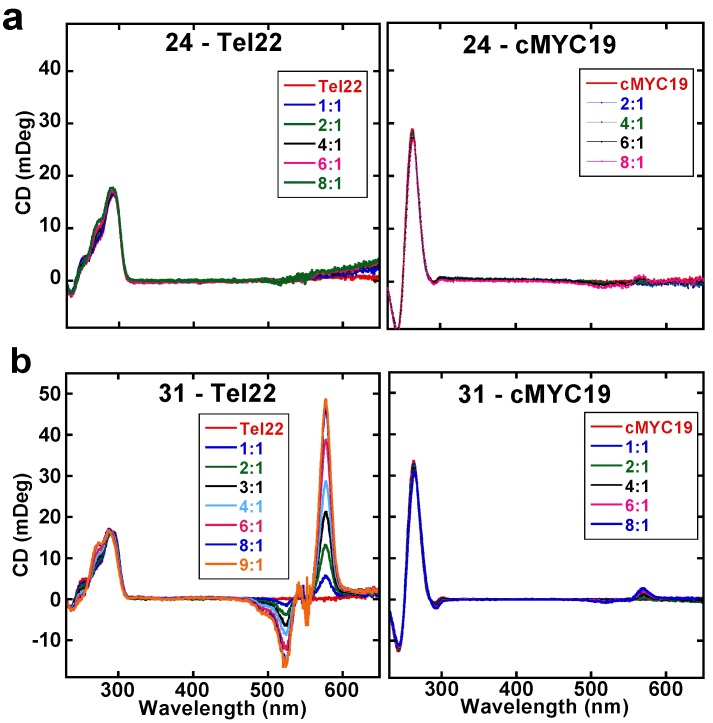
CD spectra of the parent cyanine (**24**, panel **a**) and the brominated analog (**31**, panel **b**) with Tel22 and cMYC19 quadruplex sequences. The ligands were titrated into the quadruplex solutions (5 µM) until no further change in ICD signals were obtained. The insets show the mole ratio of drug:DNA.

CD studies were conducted to qualitatively evaluate the possible binding modes of the cyanine analogs with the quadruplex structures. Upon complex formation with biomolecules, achiral ligands exhibit an induced CD (ICD) signal which is generally indicative of the binding mode of the ligand [29]. Weak ICD signals are characteristic of quadruplex end-stackers or duplex intercalators, whereas, strong ICD signals are characteristic of ligands with groove interactions [20,29]. Figure 3 shows CD spectra for **24** and **31** with both the telomeric and *c-myc* quadruplex sequences titrated until no further change in the ICD signal is observed. Compound **24** exhibits very weak ICD signals with both quadruplex sequences suggesting very weak interactions possibly by stacking at the terminal tetrads of the quadruplex. The lack of an ICD signal of **24** with Tel22 is in agreement with relatively weaker binding affinity observed in SPR and moderate increase in ∆*T*_m_ of Tel22 in screening studies. Compound **31**, however, exhibits differential ICD signals with either quadruplex sequence (Figure 3b). A large ICD signal is observed in the compound absorbance region (550–600 nm) upon complex formation with Tel22 sequence, whereas, a weak ICD signal is observed with *c-myc* quadruplex. Such large ICD signals have been observed with ligands shown to form stacked complexes in multiple grooves of the telomeric quadruplex [20]. Therefore, the strong ICD signal of **31** is possibly due to interactions in the grooves of the telomeric quadruplex. Small changes in the DNA CD signal pattern (250–300 nm) are also observed with **31** upon complex formation with the telomeric quadruplex. This is due to subtle conformational rearrangements undergone by the quadruplex structure to enhance the stacking interactions due to the additional bromine atom, and to favorably accommodate the two propyl-(trimethyl ammonium) groups in the quadruplex grooves, thereby, resulting in the strong ICD signal upon complex formation. The weak ICD signals by **31** with *c-myc* quadruplex is suggestive of a purely end-stacking mode. No change in the DNA CD signal of *c-myc* is observed suggesting that the parallel fold of the *c-myc* quadruplex is the preferred binding topology of the ligands.

### 2.4. Nuclear Magnetic Resonance Studies (NMR)

**Figure 4:**
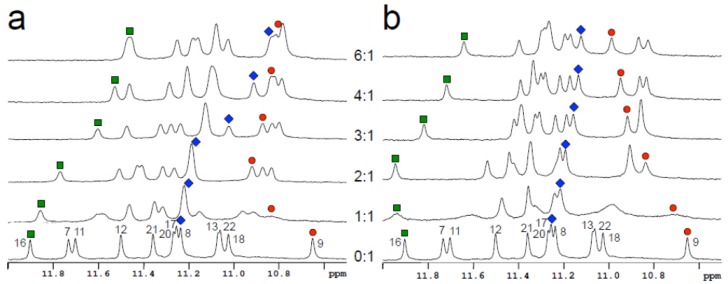
Imino proton spectra of MYC22 quadruplex titrated with the parent cyanine **24** (**a**) and the brominated analogue **31** (**b**). Ligands were added to the quadruplex at the ratio indicated on the plot. Selected imino protons of 3ʹ-end (9, red) middle (8, blue) and 5ʹ-end (16, green) tetrads are marked.

**Figure 5:**
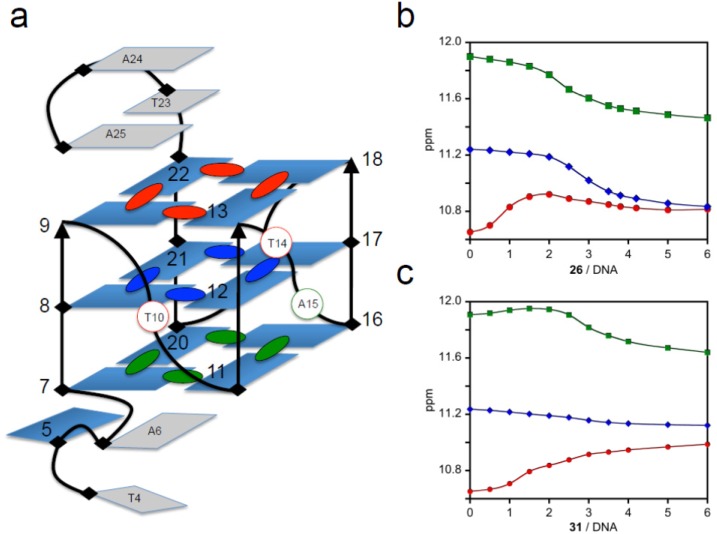
(**a**) Schematic structure of the *c-myc* quadruplex MYC22 adopted from [30]. For clarity the imino protons of the guanine tetrads are color coded. Red: 3ʹ-end tetrad, blue: center tetrad, and green: 5ʹ-end tetrad. Imino proton titration curves of 3ʹ-end, middle and 5ʹ-end tetrad for the parent cyanine (**24**, **b**) and the brominated analog (**31**, **c**).

NMR studies were carried out to locate the binding site(s) on the quadruplex structure and the imino proton spectra of a modified *c-myc* variant, MYC22, with the parent and brominated analogs are shown in Figure 4. The imino protons of MYC22 were previously assigned [30] and serve as a convenient monitor to determine the mode of interaction and stoichiometry. Based on the imino proton shifts on titration, the primary binding site for compound **24** is the 3ʹ-end tetrad of MYC22 (G9, G13, G18, and G22; Figure 4a and Figure 5b). A secondary binding site at the 5ʹ-end tetrad (G7, G11, G16, and G20) is evident after the 3ʹ-end approaches saturation. Remarkably, no precipitation was observed even at a 10-fold excess of either compound. With the exception of G8 imino proton, the middle tetrad is less affected by the binding on either terminal tetrad. However, it is noted that the shift of G8 imino proton parallels that of G16 imino proton which monitors binding to the 5ʹ-end tetrad. This suggests that the binding to the 5ʹ-end tetrad causes the chemical shift change of G8 imino proton. A similar binding sequence (first 3ʹ-end and then 5ʹ-end) is also observed for compound **31** (Figure 4b and Figure 5c). Interestingly, the presence of the bulkier bromine substituent results in a much smaller effect on the central tetrad, as shown for G8 imino proton. This is in marked contrast to the result obtained for compound **24** and indicates that there are differences in the binding orientation of **24** and **31** (on the 5ʹ-end tetrad). The NMR results, which demonstrate binding to the terminal tetrads of MYC22, are consistent with the CD results for **24** and **31** which indicate end-stacking interactions as well as small structural perturbations in the quadruplex upon complex formation. Detailed NMR studies of the proposed groove binding mode of **31** with telomeric quadruplex as observed in CD studies are currently under investigation and will be reported shortly.

### 2.5. Fluorescence Studies

**Figure 6:**
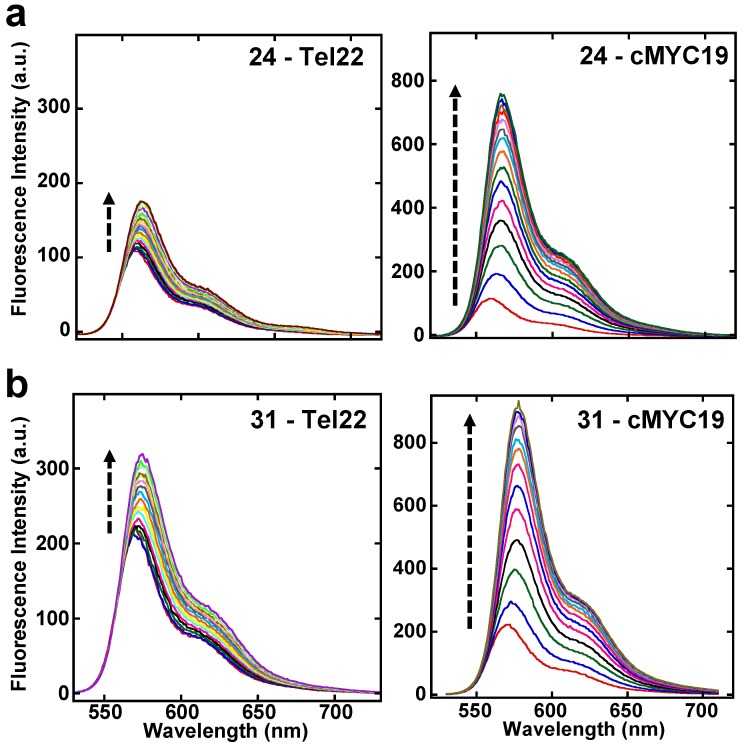
Fluorescence emission spectra of the parent cyanine (**24**, panel **a**) and the brominated analog (**31**, panel **b**) with Tel22 and cMYC19 quadruplex sequences. The arrow indicates increasing concentrations of DNA titrated into the ligand solution (1 µM) until no further change in the fluorescence emission signal was detected.

Development of fluorescence probes that are highly selective for quadruplex structures is an additional promising area given the involvement of quadruplex motifs in several important biochemical processes. The trimethine cyanines were tested for their fluorescence properties with both Tel22 and cMYC19 quadruplex structures and the fluorescence spectra of the parent cyanine **24** and the brominated analog **31** are shown in Figure 6. From the spectra, it can be readily seen that the compounds exhibit enhancement in fluorescence intensities with both quadruplex structures with increasing concentrations of DNA. However, it is strikingly apparent that the compounds exhibit very different fluorescence enhancements with both sequences. The enhancement in fluorescence intensity is significantly higher with the *c-myc* quadruplex compared to telomeric quadruplex. The preferential signal enhancement with *c-myc* quadruplex was observed with all the trimethine cyanine analogs. It is very clear that the ligands have a much stronger preference for the parallel over hybrid quadruplex. A corresponding absorbance titration of **31** with Tel22 (Figure S2) shows the presence of a single isosbestic point (~560 nm) indicating the presence of spectroscopically distinct free and bound species.

## 3. Discussion

G-quadruplex structures are associated in many important biological processes such as gene regulation and protection against chromosome degradation [3,31,32]. The recent and very exciting evidence showing the formation of G-quadruplex structures *in vivo* as well as the small-molecule mediated stabilization of these unique structures *in vivo* has further driven the need to develop potent quadruplex-targeting agents [7]. Various classes of small molecules have been developed using a G-quadruplex platform as a strategy for improved therapeutic potential as well as for enhancing the fundamental understanding of quadruplex-ligand interactions. Cyanines are one class of small molecules that have the potential as quadruplex-specific agents [33,34,35]. Previously, we have described the binding specificity and affinity of various pentamethine cyanine dyes with the telomeric and the *c-myc* oncogenic quadruplex forming sequences [18]. The pentamethine analogs exhibited a 2:1 binding stoichiometry with an end-stacking binding mode with a preferential binding for parallel *c-myc* over hybrid telomeric G-quadruplexes. The dimethyl substitutions on both indolenine rings considerably reduce their affinity for duplex systems. The 2:1 complex formation by pentamethine analogs was shown to be entropically-driven [18], as is observed for many end-stacking quadruplex binders. With the success in developing rationally designed pentamethine cyanine scaffolds for quadruplex targeting, we have extended the study with the development of a new series of cyanine molecules with reduced linker length between the aromatic cores of the cyanine scaffold. Herein, we probe the sensitivity and selectivity of binding to the polymethine chain length by shortening the distance between the heterocyclic nitrogen atoms by two carbon units.

Preliminary screening studies of the new trimethine cyanines showed considerable improvements in the thermal stability of the Tel22 quadruplex compared to the pentamethine analogs. The compounds also showed significant selectivity over duplex sequences in accord with the design principles established by the introduction of dimethyl scaffolds to reduce duplex interactions. Effectively targeting the relatively lower abundance quadruplex motifs in an essentially duplex-rich genome is a challenging task, and therefore, appropriate scaffolds with reduced duplex-DNA interactions can provide alternate platforms to develop further selective quadruplex-specific agents. This is especially true if any weak duplex interactions, such as non-specific electrostatic bindings, do not generate significant biological effects. Biosensor-SPR studies showed compound binding to different quadruplex motifs with a 2:1 binding stoichiometry and a higher preference for parallel-type quadruplex folds over hybrid quadruplexes. The more exposed terminal tetrad surface of the *c-myc* quadruplex might be more conducive for favorable stacking interactions. NMR imino proton chemical shifts provide specific evidence for cyanine stacking on both ends of the cMYC quadruplex structure. In addition, the NMR results shows that the two ends are saturated with cyanines at different ratios in agreement with the two binding affinity values (*K*_1_ and *K*_2_) observed in SPR studies. The NMR spectral changes for the parent **24** and the brominated derivative **31** suggest different stacking geometries by the two molecules. This is a very interesting result that will be very helpful in compound design as more structural details are obtained in more detailed NMR analyses.

One of the attractive features of cyanine molecules is their highly favorable fluorescence properties and their extensive application as dyes in the detection of biomolecules [36,37,38]. Benzothiazole based cyanines have been generally developed as fluorescent dyes for duplex-DNA, however, their strong interactions with duplex-DNA have limited their development as quadruplex-specific fluorescence probes. Furthermore, given the different types of quadruplex structures formed by guanine-rich sequences from various genomic regions, it is highly desirable to develop fluorescent probes that are not only selective for quadruplex structures over duplex-DNA but also discriminate between different quadruplex topologies. Very interestingly, the trimethine cyanines in the current study exhibit differential fluorescence behavior with quadruplex sequences. The significantly favorable fluorescence emission property of trimethine cyanine dyes with *c-myc* quadruplex is highly promising for their development as potential biomarkers for oncogenic-quadruplex-mediated cellular processes. The propensity of different guanine-rich sequences throughout the genome to fold into diverse quadruplex conformations will require the design of improved ligands that can discriminate among quadruplex topologies and show preferential binding with high selectivity [39,40,41]. The currently studied cyanine analogs shows many of the desirable features for targeting quadruplex structures with reduced duplex affinity as well as for discriminating different biologically important quadruplex conformations and significantly enhances our knowledge about incorporating rational design principles for selective quadruplex targeting.

## 4. Experimental

### 4.1. Synthesis of Cyanines

The chemical reagents used in the synthesis of cyanine dyes were obtained from Acros Organics (Fair Lawn, NJ, USA), Alfa Aesar (Ward Hill, MA, USA) and Matrix Scientific (Columbia, SC, USA). The reactions were followed using silica gel 60 F_254_ thin layer chromatography plates (Merck EMD Millipore, Darmstadt, Germany) with 5% methanol in DCM as the mobile phase. Open column chromatography was utilized for the purification of all final compounds using 60–200 µm, 60Å classic column silica gel (Dynamic Adsorbents, Norcross, GA, USA). The ^1^H-NMR and ^13^C-NMR spectra were obtained using high quality Kontes NMR tubes (Kimble Chase, Vineland, NJ, USA) rated to 500 MHz and were recorded on a Bruker Avance (Billerica, MA, USA) 400 MHz spectrometer using D_2_O containing tetramethylsilane (TMS) as an internal calibration standard. ^19^F-NMR spectra were recorded using hexafluorobenzene as an internal standard. UV-Vis/NIR absorption spectra were recorded on a Varian Cary 50 spectrophotometer (Palo Alto, CA, USA). High-resolution a.ccurate mass spectra (HRMS) were obtained either at the Georgia State University Mass Spectrometry Facility using a Waters Q-TOF micro (ESI-Q-TOF) mass spectrometer (Milford, MA, USA) or utilizing a Waters Micromass LCT TOF ES+ Premier Mass Spectrometer. Liquid chromatography utilized a Waters 2487 single wavelength absorption detector with wavelengths set between 640 and 700 nm depending on the dye’s photophysical properties. The column used in LC was a Waters Delta-Pak 5 µm 100Å 3.9 × 150 mm reversed phase C_18_ column. Evaporative light scattering detection analyzes trace impurities that cannot be observed by alternate methods; a SEDEX 75 ELSD (Olivet, France) was utilized in tandem with liquid chromatography to confirm purity. The preparation of the heterocyclic precursors **8**–**14** has been described by our laboratory and the compounds were used in the synthesis of the charged salts **15**–**23**.

#### 4.1.1. General Synthesis for the Alkylated Indolenine Salts **15**–**23** (Scheme 1)

Individual 2,3,3-indolenine heterocyclic precursors **8**–**14** was dissolved in anhydrous acetonitrile (50 mL) and was heated to 40 °C followed by the addition of the appropriate alkylating agent in 3 molar excess and was then heated to reflux for 72 h. The reaction progress was followed by TLC eluting with DCM/methanol/acetic acid (40/58/2). The reaction mixture was allowed to cool and the solvent was partially removed *in vacuo*. The final compounds were obtained by precipitation from methanol by diluting with acetone, ethyl acetate and diethyl ether. The hygroscopic compounds were allowed to dry for several hours under high vacuum.

#### 4.1.2. General Synthesis for the Trimethine Cyanine Binding Agents **24**–**32** (Scheme 1)

Individual alkylated indolenine salts **15**–**23** were dissolved in acetic anhydride (50 mL) followed by the addition of triethyl orthoformate in a 2:1 molar ratio. The reaction was heated at 110 °C for 30 min and followed using UV-Vis spectroscopy diluted in methanol. The mixture was allowed to cool and was crystallized using hexanes/ethyl acetate/diethyl ether. A colored precipitate was filtered, washed and collected. Compounds **24**–**32** were dried for several hours under high vacuum.

**Scheme 1:**
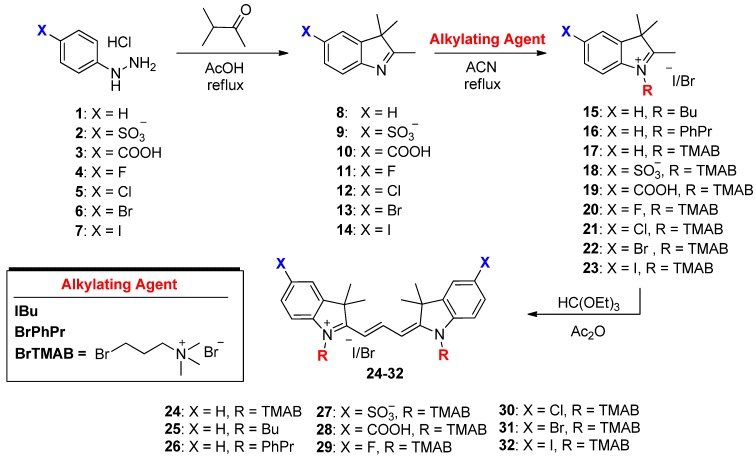
Synthetic route for the preparation of trimethine cyanine G-quadruplex binding agents.

*2-((E)-3-((E)-3,3-dimethyl-1-(3-(trimethylammonio)propyl)indolin-2-ylidene)prop-1-en-1-yl)-3,3-dimethyl-1-(3-(trimethylammonio)propyl)-3H-indol-1-ium bromide* (**24**) was obtained in 89% yield. MP 105–107 °C; ^1^H-NMR (400 MHz, D_2_O), **δ**: 1.62 (s, 12H), 2.27 (t, *J* = 7.6 Hz, 4H), 3.08 (s, 18H), 3.49–3.57 (m, 4H), 4.13 (t, *J* = 7.2 Hz, 4H), 6.52 (d, *J* = 13.2 Hz, 2H), 7.18–7.28 (m, 4H), 7.36 (d, *J* = 7.6 Hz, 2H), 7.47 (d, *J* = 7.2 Hz, 2H), 8.47 (t, *J* = 13.2 Hz, 1H). ^13^C-NMR (100 MHz, D_2_O), **δ**: 21.0, 27.3, 40.5, 49.2, 53.1, 63.2, 102.7, 110.9, 122.5, 125.7, 128.8, 140.8, 141.3, 151.8, 175.1.

*1-butyl-2-((E)-3-((E)-1-butyl-3,3-dimethylindolin-2-ylidene)prop-1-en-1-yl)-3,3-dimethyl-3H-indol-1-ium iodide* (**25**) and *2-((E)-3-((E)-3,3-dimethyl-1-(3-phenylpropyl)indolin-2-ylidene)prop-1-en-1-yl)-3,3-dimethyl-1-(3-phenylpropyl)-3H-indol-1-ium bromide* (**26**) have been previously reported by our lab and were used directly in the quadruplex binding interaction studies without any additional modifications.

*2-((E)-3-((E)-3,3-dimethyl-5-sulfonato-1-(3-(trimethylammonio)propyl)indolin-2-ylidene)prop-1-en-1-yl)-3,3-dimethyl-1-(3-(trimethylammonio)propyl)-3H-indol-1-ium-5-sulfonate bromide* (**27**) was obtained in 92% yield. MP 295–297 °C; ^1^H-NMR (400 MHz, D_2_O), δ: 1.66 (s, 12H), 2.26 (t, *J* = 6.0 Hz, 4H), 3.09 (s, 18H), 3.44–3.54 (m, 4H), 4.14 (t, *J* = 7.2 Hz, 4H), 6.44 (d, *J* = 13.2 Hz, 2H), 7.28 (d, *J* = 8.4 Hz, 2H), 7.74 (d, *J* = 8.4 Hz, 2H), 7.83 (s, 2H), 8.50 (t, *J* = 13.2 Hz, 1H). ^13^C-NMR (100 MHz, D_2_O), δ: 20.9, 21.2, 30.2, 40.8, 49.5, 53.2, 63.1, 103.7, 111.1, 120.0, 126.7, 139.9, 141.5, 143.6, 153.0, 176.4. Combustion C, H N analysis calculated for C_35_H_51_BrN_4_O_6_S_2_ (4H_2_O): C, 50.05; H, 7.08; N, 6.67. Found: C, 50.30; H, 6.83; N, 6.56.

*5-carboxy-2-((E)-3-((E)-5-carboxy-3,3-dimethyl-1-(3-(trimethylammonio)propyl)indolin-2-ylidene)prop-1-en-1-yl)-3,3-dimethyl-1-(3-(trimethylammonio)propyl)-3H-indol-1-ium bromide* (**28**) was obtained in 55% yield. MP more than 350 °C; ^1^H-NMR (400 MHz, D_2_O), δ: 1.64 (s, 12H), 2.22 (t, *J* = 6.0 Hz, 4H), 3.11 (s, 18H), 3.43–3.53 (m, 4H), 4.16 (t, *J* = 7.2 Hz, 4H), 6.46 (d, *J* = 13.2 Hz, 2H), 7.35 (d, *J* = 8.4 Hz, 2H), 7.88 (d, *J* = 8.4 Hz, 2H), 7.93 (s, 2H), 8.55 (t, *J* = 13.2 Hz, 1H). ^13^C-NMR (100 MHz, D_2_O), δ: 20.4, 21.3, 30.4, 41.8, 47.5, 54.6, 64.1, 106.7, 111.6, 122.1, 127.3, 134.9, 141.6, 147.2, 154.5, 177.4, 179.9.

*5-fluoro-2-((E)-3-((E)-5-fluoro-3,3-dimethyl-1-(3-(trimethylammonio)propyl)indolin-2-ylidene)prop-1-en-1-yl)-3,3-dimethyl-1-(3-(trimethylammonio)propyl)-3H-indol-1-ium bromide* (**29**) was obtained in 33% yield MP > 260 °C; ^1^H-NMR (400 MHz, D_2_O), δ: 1.70 (s, 12H), 2.29 (t, *J* = 7.8 Hz, 4H), 3.08 (s, 18H), 3.48 (tt, *J* = 7.8 Hz, *J* = 7.8 Hz, 4H), 4.15 (t, *J* = 7.8 Hz, 4H), 6.34 (d, *J* = 13.6 Hz, 2H), 7.14 (t, *J* = 9.2 Hz, 2H), 7.242–7.12 (m, 2H), 7.30 (d, 6.0 Hz, 2H), 8.49 (t, *J* = 13.6 Hz, 1H). ^19^F-NMR (135 MHz, D_2_O, C_6_F_6_ standard), δ: −118.8. HR-MS calculated for [C_35_H_51_N_4_F_2_Br]^+^ 644.3265, found 644.3278.

*5-chloro-2-((E)-3-((E)-5-chloro-3,3-dimethyl-1-(3-(trimethylammonio)propyl)indolin-2-ylidene)prop-1-en-1-yl)-3,3-dimethyl-1-(3-(trimethylammonio)propyl)-3H-indol-1-ium bromide* (**30**) was obtained in 77% yield. MP 255–257 °C; ^1^H-NMR (400 MHz, D_2_O), δ: 1.81 (s, 12H), 2.36 (t, *J* = 7.8 Hz, 4H), 3.29 (s, 18H), 3.95 (tt, *J* = 7.8 Hz, *J* = 7.8 Hz, 4H), 4.29 (t, *J* = 7.8 Hz, 4H), 7.21 (d, *J* = 13.4 Hz, 2H), 7.49 (d, *J* = 8.0 Hz, 2H), 7.59 (d, *J* = 8.0 Hz, 2H), 7.66 (s, 2H), 8.60 (t, *J* = 13.4 Hz, 1H). ^13^C-NMR (100 MHz, D_2_O), δ: 22.4, 28.4, 42.4, 50.9, 54.2, 64.2, 105.7, 114.0, 124.4, 130.2, 132.6, 141.9, 144.2, 153.3, 176.1. Combustion C, H N analysis calculated for C_35_H_51_Br_3_Cl_2_N_4_ (2H_2_O): C, 48.07; H, 6.34; N, 6.41. Found: C, 48.13; H, 6.31; N, 6.43.

*5-bromo-2-((E)-3-((E)-5-bromo-3,3-dimethyl-1-(3-(trimethylammonio)propyl)indolin-2-ylidene)prop-1-en-1-yl)-3,3-dimethyl-1-(3-(trimethylammonio)propyl)-3H-indol-1-ium bromide* (**31**) was obtained in 87% yield. MP 245–247 °C; ^1^H-NMR (400 MHz, D_2_O), δ: 1.71 (s, 12H), 2.16 (t, *J* = 7.4 Hz, 4H), 3.16 (s, 18H), 3.83–3.93 (m, 4H), 4.13 (t, *J* = 7.6 Hz, 4H), 7.30 (d, *J* = 13.2 Hz, 2H), 7.57 (dd, *J* = 6.4 Hz, *J* = 4.0 Hz, 2H), 7.69 (d, *J* = 6.4 Hz, 2H), 7.99 (d, *J* = 4.0 Hz, 2H), 8.38 (t, *J* = 13.2 Hz, 1H). ^13^C-NMR (100 MHz, D_2_O), δ: 21.2, 31.1, 41.6, 49.5, 53.1, 62.5, 104.5, 113.9, 118.2, 126.3, 131.8, 141.4, 151.2, 172.4, 174.2. Combustion C, H N analysis calculated for C_35_H_51_Br_5_N_4_ (3H_2_O): C, 42.84; H, 5.85; N, 5.71. Found: C, 43.09; H, 5.65; N, 5.61.

*5-iodo-2-((E)-3-((E)-5-iodo-3,3-dimethyl-1-(3-(trimethylammonio)propyl)indolin-2-ylidene)prop-1-en-1-yl)-3,3-dimethyl-1-(3-(trimethylammonio)propyl)-3H-indol-1-ium bromide* (**32**) was obtained in 92% yield. MP 260–262 °C; ^1^H-NMR (400 MHz, D_2_O), δ: 1.69 (s, 12H), 2.15 (t, *J* = 7.8 Hz, 4H), 3.15 (s, 18H), 3.88 (tt, *J* = 7.8 Hz, *J* = 8.0 Hz, 4H), 4.11 (t, *J* = 8.0 Hz, 4H), 7.28 (d, *J* = 13.4 Hz, 2H), 7.44 (d, *J* = 8.0 Hz, 2H), 7.82 (d, *J* = 8.0 Hz, 2H), 8.08 (s, 2H), 8.37 (t, *J* = 13.4 Hz, 1H). ^13^C-NMR (100 MHz, D_2_O), δ: 20.7, 27.2, 41.0, 48.8, 52.6, 62.0, 89.8, 103.9, 113.7, 131.2, 137.1, 141.4, 143.0, 150.6, 173.2. Combustion C, H N analysis calculated for C_35_H_51_Br_3_I_2_N_4_ (2H_2_O): C, 39.76; H, 5.24; N, 5.30. Found: C, 39.83; H, 5.18; N, 5.38.

### 4.2. Nucleic Acids

The DNA oligonucleotides: Tel22 d[AGGG(TTAGGG)_3_], cMYC19 d[(AGGGTGGGG)_2_A], and AATT hairpin duplex control d[CGAATTCGTTTTCGAATTCG] with and without 5’-biotin labels were purchased from Integrated DNA Technologies (Coralville, IA, USA) with HPLC purification and mass spectrometry characterization. For NMR studies, a modified *c-myc* sequence, MYC22, d[TG(AGGGTGGGG)_2_AA] was used. The concentration of all the oligonucleotides was calculated using the absorbance at 260 nm and the manufacturer provided extinction coefficients determined by the nearest-neighbor method [42]. Stock solutions of the oligonucleotides and the dyes were prepared in deionized water and diluted using appropriate experimental buffer prior to use. Experiments were conducted in 10 mM TRIS, or 10 mM HEPES for SPR, containing 50 mM KCl and 3 mM EDTA adjusted to pH 7.3.

### 4.3. UV-Thermal Melting

Thermal stability studies of the ligand-DNA complexes were performed with a Cary 300 BIO UV-visible spectrophotometer in 1 cm pathlength quartz cuvettes. Samples of ligand-DNA complexes were prepared in Tris buffer at different ratios (0:1 up to 6:1) and were mounted in a 6x6 Series II thermal block. The change in the absorbance of the complexes was monitored (at 295 nm for quadruplex samples and 260 nm for duplex samples) as a function of temperature with computer-controlled heating and cooling rates of 0.5 °C/min. A total of four scans for each sample were collected with automatic buffer correction. The buffer corrected scans were normalized and the Tm values were determined by using a combination of the derivative function and graphical estimation. The reported *∆Tm* values are averaged over two independent experiments and are reproducible within ±1 °C.

### 4.4. Biosensor-Surface Plasmon Resonance

Biosensor-SPR studies were performed with a four-channel Biacore 2000 optical biosensor system (GE Healthcare, Stockholm, Sweden) using streptavidin-derivatized sensor chips (SA). The biotinylated oligonucleotides were prepared in HEPES buffer and were immobilized using the previously described procedure [21]. A series of ligand solutions (10 nM–10 µM) prepared in HEPES buffer were injected over the DNA immobilized sensor chip at a flow rate of 25 µL/min for an association and dissociation period of 5 min each. After the dissociation phase, the sensor-chip surface was regenerated with 10 mM glycine solution (pH 2.5) for a short period and was followed by a series of buffer injections to obtain a stable baseline for the next series of ligand injections. The equilibrium steady-state response values (RU_obs_) at each injected ligand concentration (C_free_) were obtained and the values were fitted to a two-site binding model by non-linear least squares method using the following equation:

RU_obs_ = RU_max/ligand_(*K*_1_C_fre_e + 2*K*_1_*K*_2_C_free_^2^)/(1 + *K*_1_C_fre_e + *K*_1_*K*_2_C_free_^2^)

where RU_max/ligand_ is the predicted response for a single ligand binding. *K*_1_ and *K*_2_ are binding affinities for two different binding sites.

### 4.5. Circular Dichroism

CD studies were conducted using a Jasco J-810 spectrophotometer with a 1 cm pathlength quartz cuvette at 25 °C. Quadruplex-DNA solutions (4–5 µM) were annealed in Tris buffer overnight prior to the collection of spectra. An appropriate amount of the ligands were sequentially titrated into the DNA solution and the spectra were collected with a scanning speed of 50 nm/min and a response time of 1 s over the wavelength range of 230 nm to 650 nm. The spectra were collected until no further change in the induced CD signal was observed upon further ligand titration. The spectra were averaged over four scans, and a buffer scan collected in the same cuvette was subtracted from the average scan of each ratio. Data were processed and plotted using Kaleidagraph 4.0 software.

### 4.6. Fluorescence Titrations

A UV-Vis absorbance scan was obtained by scanning the ligand solutions (5 μM) in Tris buffer over 800 nm to 200 nm at a rate of 60 nm/min with a 1 nm slit width to determine their maximum excitation wavelength (λ_max_). Fluorescence experiments were performed with a Cary Eclipse Fluorescence Spectrometer at 25 °C using an excitation and emission slit widths of 5 nm and the λ_max_ of each ligand. Preannealed quadruplex DNA solutions in Tris buffer were incrementally titrated (0.05 µM) into the cuvettes containing 1 μM compound and the fluorescence emission was monitored over 500–800 nm wavelength range. Scans of the quadruplex-ligand samples were collected until no further change in the fluorescence intensity was observed upon further DNA titration. The spectra were processed and plotted with Kaleidagraph 4.0 software.

### 4.7. Nuclear Magnetic Resonance

Samples for NMR (100 µM) were prepared in 90% H_2_O/10% D_2_O and 20 mM potassium phosphate, pH 6.02. 4,4-dimethyl-4silapentane-1-sulphonic acid (DSS) was added as an internal reference. NMR experiments were performed on a Avance 600 spectrometer, equipped with a 5 mm QXI ^1^H{^31^P,^13^C,^15^N} probe (Bruker). Imino proton spectra (1024 scans) were collected at 35 °C using a WATERGATE w5 pulse sequence and a sweep width of 24 ppm.

## 5. Conclusions

Herein we are reporting the design, synthesis and characterization of novel series of trimethine carbocyanine molecules with different substituents on the indolenine rings to probe the effects on quadruplex binding. Analogous to our previous studies with penthamethine-linked cyanines, current molecules exhibit elevated quadruplex binding with considerably reduced duplex affinity. The halogen substitutions significantly increase the thermal stability and binding affinity with the quadruplex sequences with favorable preference for the parallel quadruplex motif. NMR and SPR studies show a 2:1 binding stoichiometry of the ligands while NMR and CD results support a stacking complex at the terminal tetrads of the quadruplex. The significant fluorescence enhancement observed for the parallel quadruplex over the hybrid type is a highly attractive feature of these ligands in discriminating different quadruplex folds. In summary, the current study significantly advances our knowledge in developing novel cyanine scaffolds for targeting quadruplex structures and their possible development as novel probes for G-quadruplex detection *in vivo*.

## Supplementary Materials

Supplementary materials can be accessed at: http://www.mdpi.com/1420-3049/18/11/13588/s1.
