# Supplementary material for: Selective G-Quadruplex DNA Recognition by a New Class of Designed Cyanines
Source: Molecules. 2013 Nov 4;18(11):13588–607. doi: 10.3390/molecules181113588 (PMC4457452; doi:10.3390/molecules181113588)

Supplementary Materials

Figure S1. UV-thermal melting plots of the brominated analog (31) with Tel22 (**left**) and AATT **(right)**. The melting scans were obtained at 295 nm and 260 nm for Tel22 and AATT respectively. DNA concentration is 3 μM. The insets in both plots list the drug:DNA ratios and the associated changes in thermal melting values upon complex formation are listed in Table 1.

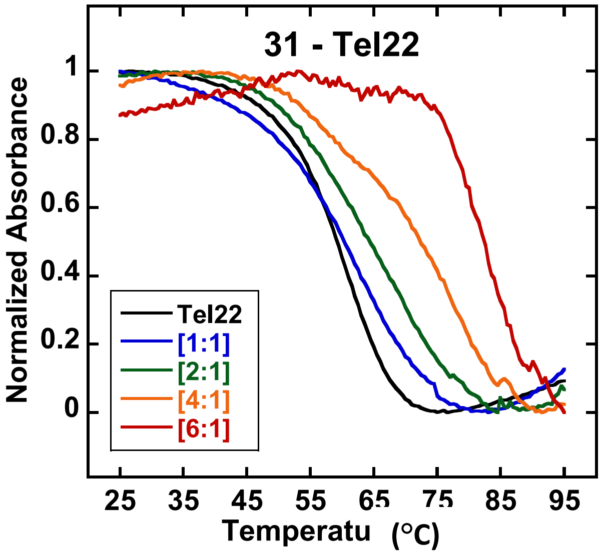

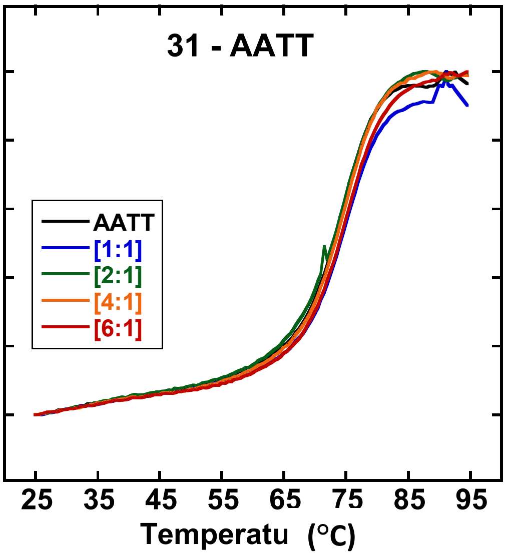

Figure S2. Absorbance titration plots of the brominated analog (31) with Tel22. The Tel22 stock solution was serially titrated into the compound solution upon no further change in the absorbance was seen. Buffer conditions for absorbance titration were same as fluorescence experiment.

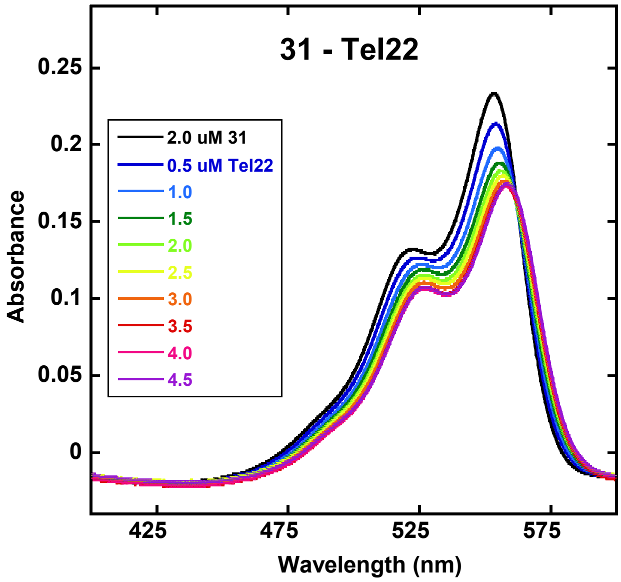

Supplement: Supplementary file 1 [file molecules-18-13588-s001.pdf]
